# The Expanding Diversity of Viruses from Extreme Environments

**DOI:** 10.3390/ijms25063137

**Published:** 2024-03-08

**Authors:** Robert D. Manuel, Jamie C. Snyder

**Affiliations:** Department of Biological Sciences, Cal Poly Pomona, Pomona, CA 91709, USA; rdmanuel@cpp.edu

**Keywords:** extremophile, archaeal virus, thermophile, acidophilic viruses, acidophile, thermophilic viruses, barophile, psychrophile

## Abstract

Viruses are nonliving biological entities whose host range encompasses all known forms of life. They are deceptively simple in description (a protein shell surrounding genetic material with an occasional lipid envelope) and yet can infect all known forms of life. Recently, due to technological advancements, viruses from more extreme environments can be studied through both culture-dependent and independent means. Viruses with thermophilic, halophilic, psychrophilic, and barophilic properties are highlighted in this paper with an emphasis on the properties that allow them to exist in said environments. Unfortunately, much of this field is extremely novel and thus, not much is yet known about these viruses or the microbes they infect when compared to non-extremophilic host–virus systems. With this review, we hope to shed some light on these relatively new studies and highlight their intrinsic value.

## 1. Introduction

**Figure 1:**
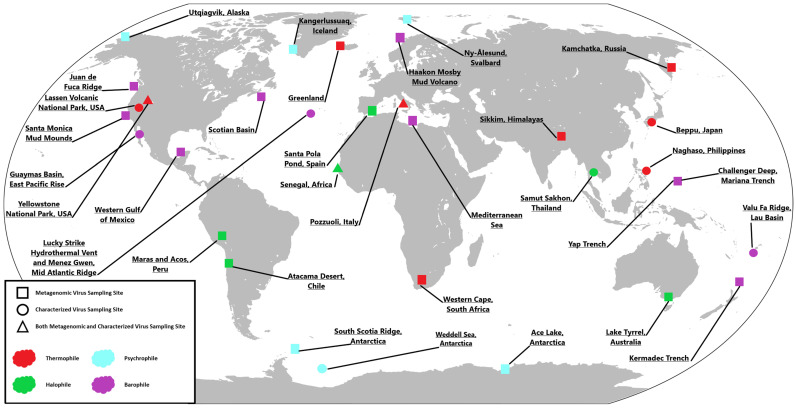
Global Sampling Map. Sampling locations from around the globe featured in this review. Both metagenomic and specific viral studies were conducted utilizing samples taken from the above locations. World map obtained from [18].

Ever since the discovery of non-living biological entities infecting tobacco plants by Dimitri Ivanovski and Martinus Beijerinck, the field of virology has expanded extensively to encompass viruses that infect all forms of life [1,2,3,4]; furthermore, viruses, being the most abundant biological entity on the planet, have been found in many different locales across the globe [5,6] (Figure 1). Historically, the study of viruses has relied on the cultivation of a host cell and the replication of the cell’s requirements within a laboratory setting. This also requires the extraction of viable virions from the environment or samples collected from the environment [7,8,9,10]. This presents its own set of challenges, as these viruses must be infectious for there to be enough viable sample to study. This viability also extends to the cells themselves as they must be able to replicate to produce viral progeny for study. Unfortunately, this can limit the scope of viral studies as many host cells are unculturable in the lab and, thus, the viruses that infect them cannot be characterized [11,12]. However, newer technological developments have allowed for the study of viruses without the need for culture-based techniques. Metagenomics has opened a whole new set of environments ripe for study by scientists [11,12,13,14,15,16,17]. Through these newer techniques, viruses from environments previously considered impossible to recreate in the lab can be studied. In this review, we focus on viruses from these extreme locales. We hope to emphasize some of the unique properties they have and shed light on some of the more novel aspects of virology.

## 2. Thermophilic/Acidophilic Viruses

Thermophilic environments are among the most well-studied extremophilic settings. Due to their relative abundance and ease of access, much research has been conducted on the organisms and viruses that inhabit these areas. Locations like Yellowstone National Park, USA, and other hot springs worldwide are relatively well studied when compared to difficult-to-reach extremophilic environments [19], such as hyperthermal deep sea vents [20,21] (Figure 1). Recently, there have been efforts to compile compendiums and reviews dedicated to cataloging these viruses [22]. However, when compared to the sheer amount of research conducted on other microbial environments, there is a need for expansion [23]. Countless viruses from these environments are still undergoing preliminary imaging to determine their structure and organizational strategy. The overall goals of many studies involve elucidating the stability of these thermophilic and acidophilic viruses in order to maintain their infectivity in such harsh conditions.

**Stability of thermophilic and acidophilic viruses.** The hyperthermophilic bacteriophage P74-26 was shown to have a significantly stable morphology due to its decoration protein gp87 (Dec^P74-26^) [24]. When compared to homologs, gp87 proves to be significantly more stable. This protein contains a core β tulip domain that is similar to the anti-CRISPR protein AcrIIC1, suggesting a common evolutionary lineage between the proteins [24]. Each subunit of gp87 has many groupings of the hydrophobic residues Isoleucine, Leucine, Valine, and Phenylalanine clustered together and buried deep within the protein [24]. Clusters of these residues have been shown to be a sign of stability in proteins [24,25]. Further analysis of this virus showed that gp87 is found to be a homotrimer that interacts with six capsid proteins through what the researchers deemed to be lasso-like interactions. These interactions between gp87 form a cage around the entire virion, which maintains the stability of the virus and functions as catch bonds. This allows expansion during genome packaging while maintaining the overall stability of the virion [26]. To this end, the authors noted that P74-26 is nearly twice as large as other T = 7 viruses. This is due to a unique loop architecture within the capsid, which creates a virion with a larger surface area while using a similar number of residues as other T = 7 virions. In addition, the main capsid protein has more complex interactions with itself and the protein gp87 cage than other *Caudoviruses* and their decoration proteins. The unusual size and structure of this capsid led the authors to hypothesize that a T = 7 capsid is the largest complexity of a viral capsid while maintaining a high degree of stability. This is mainly due to the fact that T = 7 viruses are able to maintain one type of hexon conformation, whereas T > 7 viruses must use multiple conformations. This also leads to a lower triangulation number, which minimizes the number of subunit interactions, thus reducing potential weak points within the capsid [26]. A similar thermophilic bacteriophage, P23-45, which infects the same host, *Thermus thermophilus*, was also shown to have a T = 7 capsid despite having a genome twice as large as other T = 7 viruses [27]. The authors determined that this virus is also able to increase the size of its capsid during DNA packaging while maintaining the T = 7 capsid size by increasing the size of its capsomers [27].

Conversely, the thermophilic virus *Sulfolobus* polyhedral virus 1 (SPV1) has a T = 43 icosahedral shell made up of two capsid proteins, VP4 and VP10 [28]. Researchers noted that the viral genetic material, dsDNA, is packaged in the A-form. The viral protein VP1 coats the entire dsDNA genome, forming attraction bonds between the layers of genomic material, which forms a nucleoprotein filament in the A-form [28]. This A-form genetic material coated in protein has been seen more often in filamentous thermophilic viruses such as *Sulfolobus islandicus* rod-shaped virus 2 (SIRV2) [29], *Acidianus* filamentous virus 1 (AVF1) [30], *Pyrobaculum* filamentous virus 2 (PFV2) [31], *Saccharolobus solfataricus* rod-shaped virus 1 (SSRV1), *Sulfolobus islandicus* filamentous virus (SIFV) [32], and in the novel virus.

*Sulfolobus* filamentous virus 1 (SFV1) [33]. This suggests that the A-form genetic material may be a general adaptation to extreme environments rather than an exclusive filamentous virus adaption [28,31] and that this may play a currently unknown role in stabilizing DNA within these extreme conditions [29,34]. Recently, the International Committee on Taxonomy of Viruses (ICTV) approved a new realm of classification for these archaeal filamentous viruses with A-form linear genetic material called *Adnaviria* [35]. Lastly, researchers looking at the stability of the thermophilic archaeal virus *Aeropyrum pernix* bacilliform virus 1 (APBV1) determined the 3-D structure of the virus using cryo-imaging. They utilized this to propose an assembly model and elucidate its thermostable structure [36]. APBV1 is found to contain a tight and compact capsid structure built from VP1 protein α-helices with a hydrophobic core. These VP1 proteins then interact with the supercoiled genome through Coulombic interactions, thus contributing to its thermostability [36].

**Novel thermophilic viral discoveries.** Along with examining the stability of thermophilic viruses and reporting on their structures, recent studies have elucidated some novel traits of these thermophilic viruses. Because of advancements in the field and due to hard-working, creative researchers, we are now able to study many unique characteristics of these viruses (Table 1).

*Genetic tools*. New genetic tools based on archaeal models are currently under review [42]. For example, a novel thermophilic system using the thermophilic bacteriophage P74-26 has been proposed for studying the protein structure of the small terminase protein (TerS) and its interactions with the large terminase protein (TerL) [37]. Theoretically, this model will help elucidate thermophilic viral maturation through analysis of the DNA packaging during viral assembly and the role of TerS [37]. Similarly, the thermophilic bacteriophage ΦIN93 is the focus of a study to determine its possible use as a VLP (virus-like particle) system for potential biomedical applications [38]. Specifically, the viral coat proteins ORF13 and ORF14 are of interest, as a previous study has shown that similar proteins from another thermophilic bacteriophage (VP16 and VP17 from the thermophilic bacteriophage P23-77) form complexes when expressed in vitro [38,43]. Overall, truncated versions of ORF13 and ORF14 were able to form ovoid structures that look like VLPs but are smaller than the WT virus, so further research is required [38].

*Immune evasion*. The archaeal genus *Sulfolobus* and its viruses continue to be among the most well-studied thermophilic viral systems. For example, researchers recently identified varying systems of CRISPR-Cas evasion utilized by *Sulfolobus* spindle-shaped viruses (SSVs) and *Sulfolobus islandicus* rod-shaped viruses (SIRVs), which make these viruses more infectious to their host, *Sulfolobus islandicus* [39]. Two different types of immune evasion were identified based on geographic location. One system utilized by SSVs from the volcanic hot springs in Kamchatka, Russia, involved the development of shortened genomes. These shortened genomes either lacked or rearranged the accessory genes recognized by CRISPR, thus evading host cell immunity [39]. Another system identified in SIRVs isolated from Yellowstone National Park hot springs involved mutations near the protospacers of the virus. This again prevents host immune recognition of viral genomic material, allowing an increased chance of infection [39].

*Viral assembly and egress*. Certain steps of the viral cycle of the archaeal thermophilic virus *Sulfolobus islandicus* filamentous virus (SIFV) were recently characterized [40]. During assembly, the virions are laid in a hexagonal lattice and obtain their envelope inside the cell rather than through budding. Similar to some other *Sulfolobus*-infecting viruses, pyramids form on the surface of the host cell during viral egress [40]. However, as opposed to seven-sided pyramids observed in cells infected by the turrivirus *Sulfolobus* turreted icosahedral virus (STIV) [44] and the rudivirus *Sulfolobus islandicus* rod-shaped virus 2 (SIRV2) [45], infection with SIFV results in six-sided pyramids on the surface of the cell [40]. Like c92 in STIV [46] and p98 in SIRV2 [47], the pyramids formed during SIFV infection are known to be a result of a single viral protein gp43, as the expression of this protein results in pyramid formation within *E. coli* [40]. Surprisingly, gp43 was found to contain homologs in all currently characterized viruses from viruses classified in the *Lipothrixviridae* family but not in viruses from any other families. This implies that virus-associated pyramid (VAP) proteins have undergone convergent evolution within multiple archaeal viral families [40].

Research on SSVs found that the thermophilic archaeal virus *Sulfolobus* spindle-shaped virus 9 (SSV9) has different viral release systems depending on the host being allopatric or sympatric [41]. When introduced to *Sulfolobus* host strains isolated from the same geothermal region (Kamchatka, Russia), SSV9 displays a non-lytic viral replication cycle [41]. However, when SSV9 infects *Sulfolobus* strains isolated from outside the region where SSV9 was originally discovered, the virus undergoes a lytic replication cycle [41]. The authors theorize that this may be due to a type of coevolutionary arms race, which has resulted in a cellular membrane that is more resistant to lytic cycles [41].

**Newly characterized thermophilic viruses.** New viruses from both high-temperature and low-pH environments are continuously being discovered through both traditional observational methods and newer metagenomic surveys. Due to the simplicity of replicating these environments in a laboratory, we have made great progress in isolating viruses and VLPs.

In addition, with sequencing technology rapidly advancing, we can learn more about these unique viruses in the absence of culturing techniques (Table 2).

**Figure 2:**
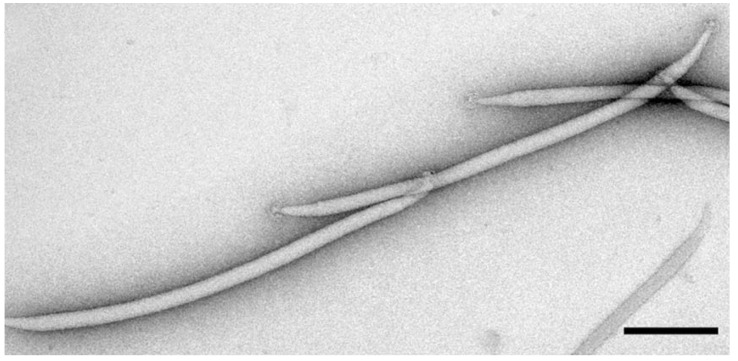
*Sulfolobus* filamentous virus 1 (SFV1). SFV1 was isolated from samples of *Sulfolobus shibatae* from the acidic hot springs of Umi Jigoku located in Beppu, Japan. The terminal mop-like structures are also visible. Some viruses were seen with elongated versions of these mop-like structures, reaching up to 700 nm in length. Reprinted from [33]. Scale bar = 200 nm.

*Culture-dependent studies*. Regarding previously characterized viruses, researchers identified and characterized a novel Fusellovirus, *Sulfolobus* spindle-shaped virus 10 (SSV10), from isolates collected from Devil’s Kitchen in Lassen Volcanic National Park, USA [48]. Further work on this viral genus identified four novel viruses deemed *Sulfolobus* spindle-shaped virus 19–22 (SSV19, SSV20, SSV21, and SSV22). These were all isolated from a hot spring located in Naghaso, Philippines [49]. SSV19 was deemed to belong to the genus *Alphafusellovirus*, while SSV20–22 all belong to the genus *Betafusellovirus*. SSV20–22 are morphologically identical, with the main differences stemming from two large variable regions within their genome. Further analysis of these *Betafuselloviruses* showed that coinfection of SSV20 and SSV22 produced a SSV21-like virus [49]. The authors theorize that a DNA recombination system present in *Sulfolobus* cells allowed for the swapping of single-nucleotide polymorphisms (SNPs) between SSV20 and SSV22 to create SSV21. Other progeny viruses may be produced in a similar manner [49]. Another virus that infects the *Sulfolobus* genus has been identified by researchers [33]. This novel virus, *Sulfolobus* filamentous virus 1 (SFV1), was isolated from the samples of *Sulfolobus shibatae* collected from the acidic hot spring Umi Jigoku located in Beppu, Japan [33]. Morphologically, this virus is 845 ± 15 nm long with an overall filamentous structure and a dsDNA genome [Figure 2]. The authors theorize an evolutionary relation to two other archaeal viruses, SIRV2 and AFV1. This theory is based on the similarities between conserved structural features of their major coat proteins rather than sequence homology [33].

Researchers recently identified five novel archaeal viruses from the active sulfurous fields of the Campi Flegrei volcano in Pozzuoli, Italy [50]. These viruses were deemed to fall within the families *Rudiviridae*, *Globuloviridae* and *Tristromaviridae* and were named *Metallosphaera* rod-shaped virus 1 (MRV1), *Acidianus* rod-shaped virus 3 (ARV3), *Saccharolobus solfataricus* rod-shaped virus 1 (SSRV1), *Pyrobaculum filamentous* virus 2 (PFV2), and *Pyrobaculum spherical* virus 2 (PSV2) [50]. Using the Genome-BLAST Distance Phylogeny methodology, the researchers proposed a new classification of the *Rudiviridae* family with six new clades of viruses [50]. Likewise, the thermophilic bacteriophage TP-84 has undergone a reclassification. As this virus has shown to have no similarities to known bacteriophages, it has been proposed to occupy a novel genus (Tp84virus) within the *Siphoviridae* family [53]. In an effort to expand on lesser-studied families of Archaea, researchers identified a novel virus, *Thermoproteus* spherical piliferous virus 1 (TSPV1), from a strain of *Thermoproteales* isolated from a hot spring in Yellowstone National Park, USA [51]. This virus has multiple 3 nm-diameter filaments extending from the surface of the virion. The average number of filaments per virion is seven, but virions have been observed to have 0–20 of these unique structures. These filaments can be up to 500 nm in length and are extremely flexible. The researchers theorize that these filaments may allow the virus to mimic the host pili, thus increasing binding probability [51].

*Culture-independent studies*. Metagenomic analyses and other newer methodologies are slowly beginning to take the forefront as culturing-based methodologies have inherit biases [11,17]. Thus, culture-independent techniques are becoming much more common. For example, researchers used both environmental metagenomics and single-cell sequencing to elucidate virus–host associations within a Yellowstone hot spring [15]. From this analysis, they found that more than 60% of the thermophilic microbes that inhabit this area contain a viral genome and that a majority contain two or more different virus types [15]. The first-ever metagenomic sampling from the hot springs of Sikkim Himalayas was conducted recently [54]. In this study, researchers identified viral sequences belonging to the *Caudovirales*, *Herpesvirales*, and *Ortervirales* orders, along with some currently unclassified giant DNA viruses [54]. The first metagenomic analysis on an African hot spring (Brandvlei hot spring) was also recently conducted [14]. Researchers were able to identify cyanophages as the dominant viral contig. As well, numerous predicted viral fragments of a *Gemmata* phage were found. Currently, there are no known *Gemmata* phages. The specific families of bacteriophages found within these samples were identified as *Myoviridae*, *Podoviridae*, and *Siphoviridae*. Regarding archaeal viruses, the researchers found the previously identified viruses His1, His2, *Acidianus* bottle-shaped virus (ABV), *Sulfolobus tengchongensis* spindle-shaped virus 2 (STSV2), and *Sulfolobus islandicus* rudivirus 3 (SIRV3) [14]. Along with this, probable virus genes were found within sequences of archaeal cells *Archaeoglobus sulfaticallidus*, *Methanobrevibacter curvatus, Methanolobus psychrophilus,* and *Methanomethylovorans hollandica* [14]. Currently, no archaeal viruses have been isolated from these species. Metagenomic analysis of six different thermophilic environments within Iceland, Yellowstone, and Italy found viruses belonging to *Ampullaviridae*, *Bicaudaviridae*, *Lipothrixviridae* and *Rudiviridae* [55]. Other researchers also utilized metagenomics to identify seven novel Uncultivated Virus Genomes (UViGs), all belonging to the *Caudovirales* order and predicted to infect the phylum *Aquificae*. From these seven UViGs, the researchers identified four “representative” viruses: *Thermocrinis* Octopus Spring virus (TOSV), *Thermocrinis* Great Boiling Spring virus (TGBSV), *Aquificae* Joseph’s Coat Spring Virus (AJCSV), and *Aquificae* Conch Spring Virus (ACSV). These viruses are proposed to belong to a novel genus *Pyrovirus* [52].

Viruses infecting hosts inhabiting thermophilic acidic environments have long been studied, and, therefore, in comparison to viruses infecting other extremophiles, we have the most information about these viruses. However, this knowledge pales in comparison to the amount of knowledge currently known about their non-extreme cousins. Often, when a new environment is sampled, we find a novel virus that expands our knowledge of these unique entities. What else will be uncovered in these investigations remains to be seen.

## 3. Halophilic/Alkalophilic Viruses

Hypersaline environments are host to microorganisms with a range of adaptations necessary for survival in extreme salinity and, often due to the chemistry of the environments, extreme alkalinity. The viruses that infect these organisms are no different. Similar to thermophilic environments, hypersaline environments are usually readily accessible to researchers worldwide [56] in the form of soda lakes [12] and other terrestrial water sources [Figure 1]. As a result, more research is conducted on these extremophiles when compared to many other extremophilic environments.

**Novel halophilic and alkalophilic viral discoveries.** Though not as researched as thoroughly as their thermophilic and acidophilic counterparts, there is much to learn from viruses infecting halophilic organisms (Table 3). For example, the physiological changes a haloarchaeal cell undergoes during infection were recently quantified [57]. *Haloarcula hispanica* was infected with the halophilic icosahedral internal membrane-containing SH1, icosahedral tailed HHTV-1, spindle-shaped His1, and pleomorphic His2 viruses [57]. The cell was monitored for oxygen consumption, binding of the lipophilic anion phenyldicarbaundecaborane, and ATP levels inside and outside the cell [57]. Through this, they were able to determine that SH1 and HHTV-1 induced lysis within the host cell while His1 and His2 were seen to be non-lytic [57]. These results appear to be similar to the thermophilic SSVs mentioned above in that infection of a cell by similar viruses can yield different viral life cycles.

In order to study viral lysis, researchers utilized an unbiased mutation approach of ORF79 within the haloalkaliphilic virus θCh1 and elucidated a repressor function. Without ORF79 functioning correctly, lysis and viral protein expression occurred prematurely [58]. The overexpression of this ORF entailed the absence of lysis and a complete lack of viral proteins/progeny [58]. Similar research utilized mutation-based assays to determine that several open reading frames (ORF4 and ORF11-12) were necessary for the replication and regulation of the halophilic virus SNJ1 [59]. This virus was shown to utilize rolling circle replication based on the accumulation of single-stranded replicative intermediates [62]. Furthermore, researchers identified that a family of PL6 and PL6-like plasmids within the halophilic microorganism *Haloquadratum walsbyi* and metavirome data are relatively conserved among known haloviruses. These plasmids have been shown to be significantly related to each other, and a variety of viruses are known to infect these halophiles. For example, the protein F3 found in these plasmids is strongly related to open reading frame 9 within the betapleolipovirus HRPV-3 [60]. Likewise, it has been seen to be related to other haloviruses, such as HGPV-1 and SNJ1 [60,61]. These plasmids have been found to be highly conserved in samples from distinct locations, indicating that these plasmids may be widespread in halophilic organisms [60].

Technological limitations and environmental factors often prevent extremophilic viruses from having wild-type host–pathogen interactions within a laboratory setting [63]. One study found some success using asymmetrical flow field-flow fractionation to keep the infectivity of halophilic viruses while also purifying them from various substrates and media [63]. This may be a promising avenue regarding the filtration and extraction of viable viruses from samples, as researchers are able to use this trait as a way to retain viable virions on a standard microfilter [64]. Studies like this, however, are in their infancy when it comes to extremophiles. When studying specific viruses, researchers are usually limited to the technology and environmental factors easily replicated within a laboratory setting. Another study found that some halophilic viruses may utilize a type of quorum sensing through ionic strength detection [64]. When sodium ions within a solution were lowered, T4 phages were found to aggregate together. The researchers suggest that this may be an evolutionary mechanism to promote the survival of the phage while outside the host [64]. In comparison to non-extremophilic settings, not much is known about these halophilic systems. Additional research on these viruses may eventually lead to genetic systems and a better understanding of these high-salinity microbial environments [65].

**Figure 3:**
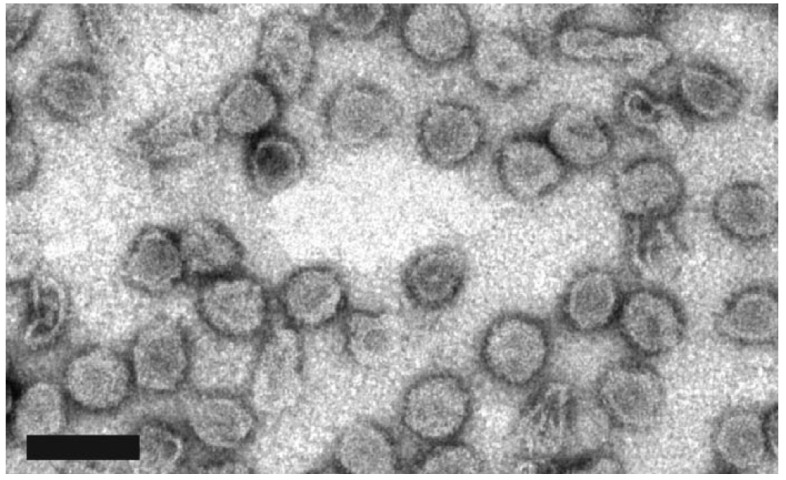
*Halorubrum* pleomorphic virus 12 (HRPV12). The tailless, round particles of HRPV12 resemble other pleolipoviruses. These viruses were isolated from Lake Retba near Senegal, Africa. This virus was seen to only infect two different species of the halophilic microorganism *Halorubrum*, suggesting a very narrow host range. Further analysis of HRPV12 indicated that it is a member of the genus *Betapleolipovirus*. Reprinted with permission from [8]. Copyright 2019 John Wiley and Sons. Scale bar = 100 nm.

*Culture-dependent studies.* Many novel halophilic viruses are still the subject of further characterization (Table 4). Several new *Pleolipoviridae* viruses (*Haloarcula hispanica pleomorphic* virus 4 [HHPV4] [66], *Halorubrum pleomorphic* virus 9 [HRPV9] [67], HRPV10, HRPV11, HRPV12 (Figure 3), and one new *Caudovirales* designated *Haloferax* tailed virus 1 (HFTV1) [8] have recently been described. This family of viruses is distributed globally and includes both single and double-stranded DNA viruses [68]. Additionally, a novel myovirus designated ChaoS9 (Chao: Caudovirus of haloarchaeal origin; S9) was recently characterized [69]. They proposed that this novel virus would best fit within the genus *Myohalovirus* as its head and tail proteins are similar to other halophilic viruses, phiH1 and phiCh1, within this genus [69].

Sometimes, newly discovered viruses can be in flux when it comes to classification. More often than not, new information can lead to reclassification and a better understanding of viral familial relations. For example, the halophilic virus *Haloarcula californiae* icosahedral virus 1 (HCIV-1) has been proposed to be reclassified to the genus *Alphasphaerolipovirus* based on its similarities to other tailless icosahedral internal membrane-containing haloarchaeal viruses within this genus [71]. This virus has also been visualized via cryo-electron microscopy along with *Haloarcula hispanica* icosahedral virus 2 (HHIV-2) at 3.7 and 3.8 Å resolution. This revealed the organizational method behind these vertical single β-barrel viruses [72]. Another cryo-electron microscopy-based study was conducted on the halophilic virus SH1. This study elucidated the evolutionary link between SH1-like viruses with a double β-barrel virus lineage, which includes the largest known giant viruses [73].

*Culture-independent studies.* Recently, metagenomic studies have been used to identify novel viruses within these halophilic communities [12]. One such study on halophilic viruses found that these viral communities differ along a salinity concentration gradient [74]. *Caudovirales* were found to be the most persistent regardless of salinity level around the globe. Low (4–15%) and high (22–37%) salinity environments were also seen to have similar patterns based on the specificity of viral–host interactions [74]. Another study looked at the halophilic viruses found within the high-altitude hypersaline pools in the Peruvian Andes [75]. Utilizing sequencing and metagenomic data sets, the researchers identified viral sequences from the *Caudovirales*, *Adenovirus, Herpesvirus*, *Phycodnaviridae*, *Poxviridae*, *Mimiviridae*, and *Pandoravidae* families [75]. Additionally, an unclassified group of archaeal dsDNA viruses with a spindle-shaped morphology was found [75]. This spindle-shaped morphology is prevalent in thermophilic archaeal viruses, indicating that there is much more within these environments ripe for study [76].

Halophilic viruses from halite nodules within the Atacama Desert were seen to consist of the families *Caudovirales*, *Pleolipoviridae*, and *Sphaerolipoviridae*. These viruses were seen to infect primarily *Halobacteria* and *Salinibacter* hosts [77]. However, many of these viruses had no detectible transcription within most samples [77]. This often happens with viral extracts from extreme environments, as a number of currently unknown factors can play a role in the production of viral progeny.

## 4. Psychrophilic Viruses

It is well documented that many viruses exhibit the ability to remain virulent after exposure to extremely frigid temperatures. However, the viruses that thrive and infect other microbes at these low temperatures are currently undescribed. Little research has been conducted on these extremophilic microorganisms and their viruses, and what has been studied is biased based on location [78] (Figure 1).

**Usage of psychrophilic enzymes.** Recently, there has been a growing interest in the properties of the microorganisms and viruses that inhabit low-temperature environments, particularly in multiple industries [79]. Ice-binding proteins (IBPs) from these extremophiles can be used in food products to preserve quality in freezers or other low-temperature preservation methods [80]. IBPs can also be used within medicinal fields to store biological materials safely at low temperatures without compromising cells [80]. Along with IBPs, low-temperature active enzymes are of note. The lack of energy input needed to kickstart reactions with psychrophilic enzymes is what drives much of this research. These enzymes function best at moderate temperatures and thus negate the need for drastic energy input [81]. Their applications range from medicinal to more commercial avenues. Detergents are one such example that can benefit from low-temperature enzymes. These have the potential to improve the efficiency of cleaning by saving on energy costs and efficiently removing stains at lower temperatures [81,82]. Cold-activated enzymes even have uses within the dairy industry, and β-D-Galactosidases have been used to produce lactose-free products [80,83]. Overall, there is great potential in this industry; however, there needs to be an expansion of research into these microorganisms, especially the viruses that infect them.

**Novel psychrophilic viral metagenomic studies.** Though novel viruses can still be isolated from arctic ice through traditional means [84], metagenomics seems to be the preferred method of analysis in these environments (Figure 1). While still being optimized, this type of research has provided scientists with the opportunity to identify non-culturable viruses and their hosts from these unique environments [16]. It is known that there is a wealth of both DNA and RNA viruses within these settings [85]. However, many of these viruses appear to be completely unique to these environments. For example, melting permafrost soil samples from northern Sweden showed that only 15% of viruses could be assigned a taxonomy [86].

*Cryoconite hole and cryopeg viruses.* Within cryoconite holes from Svalbard and the Greenland Ice Sheet, researchers identified numerous viruses based on circular genome scaffolds (CGSs) [87]. They then placed these CGSs into 12 novel groups based on whole-genome comparisons [87]. When analyzing the data, the authors noted varying survival strategies based on their sequence analysis [87]. Of these CGSs, 49 contained integrase genes, 25 contained ParA/B systems, 3 contained toxin–antitoxin systems, and 8 contained a putative satellite phage group like the phage P4 [87].

A metagenomic study on the viruses that exist in cryopegs, sea-ice brine, and bulk sea ice near Utqiaġvik, Alaska, found 476 unique viral populations [88]. Of these virus populations, 221 shared little to no viral genes with known viruses, and only 56 could be assigned to a described group of viruses. There seem to be distinct viral communities between the cryopeg and sea-ice environments, with the cryopeg being generally more diverse [88]. The identification of fatty acid desaturase (FAD) genes led the authors to hypothesize that these psychrophiles utilize FAD genes to help their hosts survive in these frigid conditions [88]. Another study on the RNA viruses within permafrost identified eight different phylogenetic clades of viruses [89]. These viruses are correlated with environmental factors and eukaryotes and may have a great influence on microbial community dynamics and metabolic function [89].

Analysis of a cryopeg near Utqiaġvik, Alaska (called Barrow in the manuscript) found that 95% of the viruses located in this area were dsDNA, with 85% coming from the order *Caudovirales* [90]. Of these, over 40% had similarity to 13 different viruses, which included multiple *Bulkholderia* phages, *Loktanella* phage pCB2051-A, deep sea thermophilic phage D6E, *Providencia* phage Redjac, *Enterobacter* phage Enc34, *Pseudomonas* phage YuA, *Clostridium* phage phi3626, *Pseudomonas* phage B3, *Salmonella* phage FSL SP-088, *Bacillus* phage PBC1 and *Nitrincola* phage 1M3-16 [90].

*Caudovirales* seem to be the dominant order of viruses from this type of environment. A study on the effect of polar light on Antarctic Lake dynamics found that viral abundance decreased with depth [91]. Analysis found 173 unique viruses, most of which belonged to the *Caudovirales* order [91]. Likewise, metagenomic analysis of viruses from the South Scotia Ridge near the Antarctic found that *Caudovirales* were again the most common order, with the families *Podoviridae*, *Siphoviridae*, and *Myoviridae* being the most abundant [92]. This is similar to previous studies in the arctic regions [92]. However, some studies on viruses from permafrost are thought-provoking.

**Figure 4:**
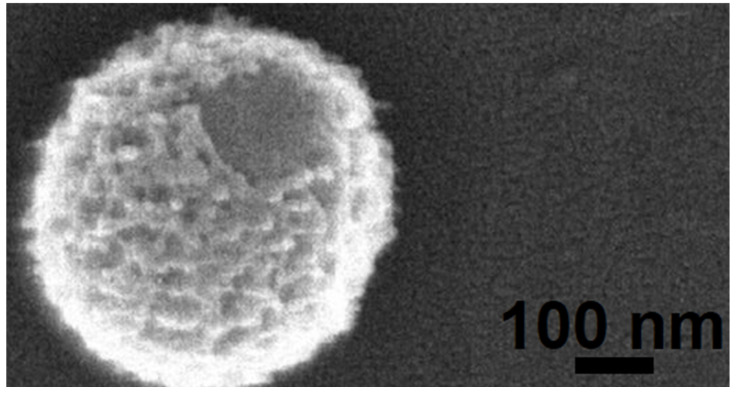
***Mollivirus sibericum***. This giant psychrophilic virus was isolated from the same 30,000-year-old permafrost sample as *Pithovirus sibericum* [93] from Chukotka, Russia. The virus is estimated to be as old as the permafrost itself and yet retains its virulence. Infection of the amoeba *Acanthamoeba castellanii* was seen under microscopy. Reprinted with permission from [94]. Copyright 2015 C. Abergel and J.M. Claverie. Scale bar = 100 nm.

**Viral preservation in permafrost.** With the discovery of multiple viable 30,000-year-old giant viruses from Siberian permafrost [93,94] (Figure 4) and 700-year-old preserved viral genomes within caribou feces [95], some scientists now consider the melting permafrost to be an ecological ticking time bomb [96,97]. As mentioned above, many viruses are continuously being discovered in these freezing areas, of which a significant portion have no known taxonomy [86,87,88,91]. While none have currently been identified, some unknown viruses may include a select few that can harm humanity [98,99,100]. These viruses are as old as the permafrost they exist in [101]. To modern environments, these are essentially novel pathogens [100]. This, coupled with melting permafrost, can lead to a significant issue as these viruses and other possible contaminants can have a drastic effect on the already stressed polar ecosystem [97]. While studies on the microbes that exist in this environment have been picking up steam [102], there is a distinct lack of research on the viruses from these frigid environments [103].

## 5. Barophilic Viruses

One of the most remote and unexplored areas of modern science is the deep oceanic biome. Due to modern technological advancements, the microbes and viruses that exist in this area have only recently been accessible to researchers [104] (Figure 1). As a result, little is known about the viruses that inhabit this area and the microbes that they infect. However, it is theorized that viruses play a key role in the biodiversity of microorganisms and the cycling of organic materials within the ocean [105,106]. In the hadal regions of the ocean floor, viruses play a major role in prokaryote biomass production within these remote areas [107,108,109,110]. It is estimated that 0.3–0.5 gigatons of carbon is cycled alone from barophilic lytic viruses infecting archaeal cells in hadal regions annually [111]. Interest in this area is growing as a possible resource for new types of hydrolases that can withstand extreme conditions [112]. However, this research is still in its infancy [113], and the focus is specific to microbes of the deep and not the viruses that infect them. However, it has been shown that viruses from these environments may harbor significantly useful enzymes as well [114].

**Figure 5:**
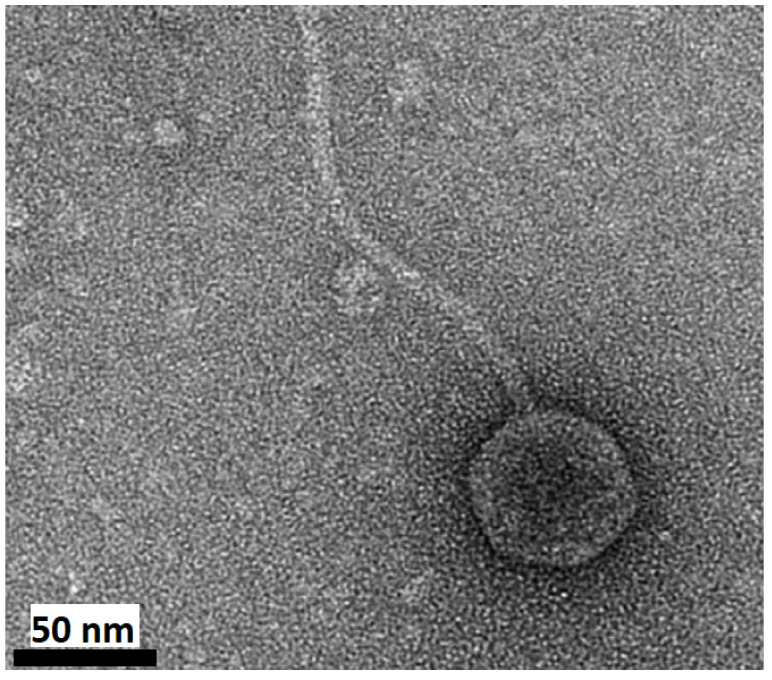
*Marinitoga piezophila* virus 1 (MPV1). This *Siphoviridae*-like virus was isolated from a strain of *Marinitoga piezophila*, a thermophilic microbe found on deep sea hydrothermal vents on the East Pacific Rise. Along with its own genetic material, this virus packages a plasmid-based mobile genetic element from its host, 13.3 kb in size. Reprinted with permission from [21] Copyright 2015 Elsevier. Scale bar = 50 nm.

**Culture-dependent studies.** Only 11 viruses have been characterized from abyssal environments [20,21,115] (Figure 5), and of those, only one has been described as infecting deep-sea animals [116]. Of these characterized viruses, many come from hydrothermal vent locations around the world (Figure 1). For example, researchers identified two novel deep-sea barophilic viruses, *Marinitoga camini* virus 1 and 2 (MCV1 and MCV2), from hydrothermal vents in the Mid-Atlantic Ridge [115]. These viruses infect the *Marinitoga* genus of bacteria and can be placed in the *Siphoviridae* family based on their morphology [115]. Likewise, *Methanocaldococcus fervens* tailed virus 1 (MFTV1) was identified from deep-sea hydrothermal vents on the East Pacific Rise and the Mid-Atlantic Ridge [20]. This virus has been shown to infect multiple strains of *Methanocaldococcus*, and morphological traits classify this virus in *Siphoviridae* [20]. While not necessarily a characterization, genomic analysis of a novel thermophilic heterotrophic anaerobe, *Marinitoga lauensis*, from deep-sea hydrothermal vents near the Eastern Lau Spreading Center and Valu Fa Ridge revealed a novel prophage [117]. This prophage was found to be similar to the characterized deep-sea viruses MCV1, MCV2, MPV1 and *Marinitoga* sp. 1137 [117]. Analysis of the characterized deep-sea virion GVE2 [118] has shown that it encodes a thermostable His-Asn-His (HNH) endonuclease [119,120] and that its tail spike protein has unique traits along with the ability to slow down the growth of its host cell *Geobacillus* [10].

**Culture-independent studies.** While it is a new field, especially in this biome, researchers are currently refining the methodology used to isolate viral genetic information from deep-sea regions [13] (Figure 1). Like other extremophilic viruses, one major way that researchers study them is through metagenomic analysis. One such study from two deep-sea sites in the Mediterranean identified 99 contigs of obvious viral origin, with 75 belonging to the *Caudovirales* order [121]. Of these 75, many were completely novel viruses, exclusive to the mesopelagic and bathypelagic regions of the ocean and as widespread as known surface viruses [121]. Similarly, a study on the Challenger Deep of the Mariana Trench found that only 24–30% matched known sequences. Of the viral sequences, the families *Microviridae*, *Circoviridae*, *Nanoviridae*, and *Geminiviridae* were found to be most prevalent [122]. Another metagenomic study in the same location found 15 different viral families, with most belonging to the *Caudovirales* order. The dominant families included *Myoviridae, Siphoviridae*, and *Podoviridae* [123]. The researchers also identified auxiliary metabolic genes (AMGs) within a number of viruses that altered the host’s sulfur and nitrogen metabolism, thus providing more evidence of the virus’ role within the nutrient cycling of these deep-sea regions [123].

Sampling from multiple deep sea drilling sites near the Juan de Fuca Ridge found viruses with morphological similarities to the order *Caudovirales, Bicaudaviridae* family, *Fuselloviridae* family, *Rudiviridae* family, untailed viruses, filamentous viruses, spindle-shaped viruses, and a bilobate like structure that could possibly be a novel viral morphology [124]. Of all drilling sites sampled, 60–80% were predicted to have archaeal hosts. Metagenomic analysis showed that *Myoviridae* and *Siphoviridae* were the dominant families as well as currently unclassified tailed archaeal viruses [124]. Analysis of 19 different metagenomes from seawater and sediment samples of the Mariana, Yap, and Kermadec Trenches found that many viruses from these areas are unclassified and unsampled [125]. Of the viruses able to be matched, *Myoviridae*, *Siphoviridae*, and *Podoviridae* were again the most dominant families, with many of these viruses concurrent throughout the three different trenches sampled. Surprisingly, 77 of the virus operational taxonomic units (vOTUs) identified were classified as nucleocytoplasmic large DNA viruses, with two having over 200 kb size genomes [125]. Like a previously mentioned study [123], these researchers also identified viral AMGs that are able to alter the host’s metabolism [125]. Another metagenomic sampling from seven different cold seeps from around the world (Haakon Mosby mud volcano, Eastern North Pacific Ocean, Mediterranean Sea, Amon mud volcano, Santa Monica Mounds, Eastern Gulf of Mexico, Scotian Basin, and Western Gulf of Mexico) also showed similar results [126]. Herein, 2885 vOTUs were identified from these sites, with 4 being over 200 kb in length. Of these viruses, 96% were unclassifiable, with the remaining 4% coming from the families *Podoviradae*, *Myoviradae*, and *Siphoviradae*, with their hosts consisting of bacteria and archaea. Likewise, AMGs were found in some of the viral OTUs related to the carbon, sulfur, and nitrogen metabolism of the host cells. Contrasted to previously mentioned studies, these viruses sampled from cold seeps were seen to have a high degree of endemism both in each sampled site and overall. Of these vOTUs, 78.7% are found in no other location on Earth [126].

## 6. Conclusions

While there have been great strides in recent years, the field of extreme virology remains a small subsection of virology. However, there has been much progress in the field within the past few years. With the development of new technology and increased interest within these environments, we imagine that there will be many more novel discoveries in the near future. Like their hosts, we believe these viruses have much potential in multiple fields, i.e., academic, industrial, and humanitarian. From an academic perspective, these viruses provide many unique morphologies never seen in their non-extremophilic counterparts. These traits are a result of millions of years of adaptations to both exist and infect their respective hosts in extreme environments. Extremophilic adaptations are fascinating, and much of the specifics remain a mystery. However, as the fields of proteomics and metagenomics continue to develop, we believe that common trends will be observed among viruses from similar extreme environments. In addition, we believe that the study of extremophilic viruses may also provide possible industry applications. Like their hosts, these viruses must exist in these environments and must remain stable to be virulent. As mentioned above, enzymes sourced from psychrophilic and thermophilic cells are currently of great use in industry. Viruses from other extreme environments may be an untapped well of potential for commercialization. Lastly, from a humanitarian viewpoint, there may be valid concerns from a subsection of these extremophilic viruses, mainly psychrophiles. As stated above, there have been recent examples of viable viruses preserved within the melting permafrost. Likewise, many of these environments contain viruses unknown to modern science. While most are indeed phages and noninfectious to humans, many of these viruses can be considered novel to modern-day environments. It is unknown what type of effect they may have within an environment that has lacked their presence for tens of thousands of years. As of now, we can only speculate.

Overall, much is still unknown about the virosphere from these extreme environments, in particular, the more inaccessible regions such as the abyssal and arctic. Only recent studies have highlighted a few viruses from these areas. Hopefully, by shedding some light on the more recent work in this field, more interest will be drawn to these fascinating members of the virosphere. From unraveling the structural stability of thermophilic and acidophilic virions to identifying and categorizing newly emerging viruses from melting permafrost, extremophilic virology has major research potential.

## Figures and Tables

**Table 1:** Thermophilic and acidophilic viral discoveries.

Virus	Discovery	Source
P74-26	Novel thermophilic system developed for studying the small terminase protein (TerS) structure and interactions with large small terminase protein (TerL).	[37]
ΦIN93	Viral coat proteins ORF13 and ORF14 have potential to be used as a VLP system to deliver medications.	[38]
*Sulfolobus* spindle-shaped viruses (SSVs)	Utilized shortened genomes to truncate the accessory genes recognized by host CRISPR, thus evading host immunity.	[39]
*Sulfolobus islandicus* rod-shaped viruses (SIRVs)	Contain mutations near protospacers to elude recognition by host CRISPR, again evading host immunity.	[39]
*Sulfolobus islandicus* filamentous virus (SIFVs)	Infection causes the formation of unique six-sided pyramids on the surface of the host. These pyramids are the result of a single protein, gp43.	[40]
*Sulfolobus* spindle-shaped virus 9 (SSV9)	Has a varying viral egress strategy depending on the host being allopatric or sympatric to the region SSV9 was discovered.	[41]

**Table 2:** Newly characterized thermophilic and acidophilic viruses.

Virus	Viral Family/Genus	Location Isolated	Source
*Sulfolobus* spindle-shaped virus 10 (SSV10)	*Fuselloviridae*	Devil’s Kitchen in Lassen Volcanic National Park, USA	[48]
*Sulfolobus* spindle-shaped virus 19 (SSV19)	*Alphafusellovirus*	Naghaso, Philippines	[49]
*Sulfolobus* spindle-shaped virus 20 (SSV20)	*Betafusellovirus*	Naghaso, Philippines	[49]
*Sulfolobus* spindle-shaped virus 21 (SSV21)	*Betafusellovirus*	Naghaso, Philippines	[49]
*Sulfolobus* spindle-shaped virus 22 (SSV22)	*Betafusellovirus*	Naghaso, Philippines	[49]
*Sulfolobus* filamentous virus 1 (SFV1)	Unknown	Acidic hot spring Umi Jigoku in Beppu, Japan	[33]
*Metallosphaera* rod-shaped virus 1 (MRV1)	*Rudiviridae*	Active Sulfurous Fields of the Campi Flegrei volcano in Pozzuoli, Italy	[50]
*Acidianus* rod-shaped virus 3 (ARV3)	*Rudiviridae*	Active Sulfurous Fields of the Campi Flegrei volcano in Pozzuoli, Italy	[50]
*Saccharolobus solfataricus* rod-shaped virus 1 (SSRV1)	*Rudiviridae*	Active Sulfurous Fields of the Campi Flegrei volcano in Pozzuoli, Italy	[50]
*Pyrobaculum* filamentous virus 2 (PFV2)	*Rudiviridae*	Active Sulfurous Fields of the Campi Flegrei volcano in Pozzuoli, Italy	[50]
*Pyrobaculum* spherical virus 2 (PSV2)	*Rudiviridae*	Active Sulfurous Fields of the Campi Flegrei volcano in Pozzuoli, Italy	[50]
*Thermoproteus* spherical piliferous virus 1 (TSPV1)	*Globuloviridae*	Yellowstone National Park, USA	[51]
*Thermocrinis* Octopus Spring virus (TOSV) *	*Pyrovirus*	Octopus Spring, WY	[52]
*Thermocrinis* Great Boiling Spring virus (TGBSV) *	*Pyrovirus*	Great Boiling Spring, NV	[52]
*Aquificae* Joseph’s Coat Spring virus (AJCSV) *	*Pyrovirus*	Joseph’s Coat Spring, WY	[52]
*Aquificae* Conch Spring virus (ACSV) *	*Pyrovirus*	Conch Spring, WY	[52]

* Derived from metagenomic data.

**Table 3:** Halophilic/alkalophilic viral discoveries.

Virus	Discovery	Source
SH1	Induces lysis within host cell *Haloarcula hispanica*	[57]
HHTV-1	Induces lysis within host cell *Haloarcula hispanica*	[57]
His1	Non-lytic life cycle within host cell *Haloarcula hispanica*	[57]
His2	Non-lytic life cycle within host cell *Haloarcula hispanica*	[57]
θCh1	ORF79 is required for correctly timed viral lysis and protein expression	[58]
SNJ1	ORF4 and ORF11-12 are necessary for replication and regulation	[59]
PL6 and PL6-like plasmids	Show significant relatedness to the halophilic viruses HRPV-3, HGPV-1, and SNJ1	[60,61]

**Table 4:** Newly characterized halophilic viruses.

Virus	Viral Family/Genus	Location Isolated	Source
*Haloarcula hispanica* pleomorphic virus 4 [HHPV4]	*Pleolipoviridae*	Unknown *	[66]
*Halorubrum* pleomorphic virus 9 [HRPV9]	*Pleolipoviridae*	Samut Sakhon, Thailand	[67,70]
HRPV10	*Pleolipoviridae*	Lake Retba, Senegal	[8]
HRPV11	*Pleolipoviridae*	Lake Retba, Senegal	[8]
HRPV12	*Pleolipoviridae*	Lake Retba, Senegal	[8]
*Haloferax* tailed virus 1 (HFTV1)	*Caudovirales*	Lake Retba, Senegal	[8]
Caudovirus of haloarchaeal origin; S9 (ChaoS9)	*Myohalovirus*	Unknown **	[69]

Newly characterized halophilic viruses. * Viruses isolated from lab stock that were randomly lysed. ** Origin not listed in publication.

## Data Availability

No new data were created or analyzed in this study. Data sharing is not applicable to this article.
